# Effects of tDCS on Bimanual Motor Skills: A Brief Review

**DOI:** 10.3389/fnbeh.2018.00063

**Published:** 2018-04-04

**Authors:** Nils H. Pixa, Bettina Pollok

**Affiliations:** ^1^Department of Sport Psychology, Institute of Sports Science, Johannes Gutenberg-University Mainz, Mainz, Germany; ^2^Institute of Clinical Neuroscience and Medical Psychology, Medical Faculty, Heinrich-Heine-University Düsseldorf, Düsseldorf, Germany

**Keywords:** non-invasive brain stimulation, transcranial direct current stimulation, bimanual movements, bimanual coordination, motor learning and performance

## Abstract

Transcranial direct current stimulation (tDCS) is a non-invasive brain stimulation technique that allows the modulation of cortical excitability as well as neuroplastic reorganization using a weak constant current applied through the skull on the cerebral cortex. TDCS has been found to improve motor performance in general and motor learning in particular. However, these effects have been reported almost exclusively for unimanual motor tasks such as serial reaction time tasks, adaptation tasks, or visuo-motor tracking. Despite the importance of bimanual actions in most activities of daily living, only few studies have investigated the effects of tDCS on bimanual motor skills. The objectives of this review article are: (i) to provide a concise overview of the few existing studies in this area; and (ii) to discuss the effects of tDCS on bimanual motor skills in healthy volunteers and patients suffering from neurological diseases. Despite considerable variations in stimulation protocols, the bimanual tasks employed, and study designs, the data suggest that tDCS has the potential to enhance bimanual motor skills. The findings imply that the effects of tDCS vary with task demands, such as complexity and the level of expertise of the participating volunteers. Nevertheless, optimized stimulation protocols tailored to bimanual tasks and individual performance considering the underlying neural substrates of task execution are required in order to probe the effectiveness of tDCS in greater detail, thus creating an opportunity to support motor recovery in neuro-rehabilitation.

## Introduction

The non-invasive brain stimulation technique of transcranial direct current stimulation (tDCS) is a suitable method for modulating cortical excitability (Bindman et al., 1964; Nitsche and Paulus, 2001) as well as neuro-plastic reorganization (Fritsch et al., 2010; Hunter et al., 2013; Karabanov et al., 2015). Previous studies suggest that tDCS can be used to facilitate motor performance such as motor learning in healthy volunteers (Reis and Fritsch, 2011; Buch et al., 2017) and in patients suffering from neurological disorders (Bastani and Jaberzadeh, 2012; Flöel, 2014). Notably, the effects of tDCS on motor performance are almost exclusively evidenced by studies employing unimanual tasks. However, it is well known that in a large variety of daily activities both hands are required to accomplish required actions. Impaired bimanual skills due to neurological disease or age-related decline (for an overview see Maes et al., 2017) present a challenge to independent living. Despite the abundance of daily activities that require bimanual skills, only few studies have thus far investigated the effects of tDCS on bimanual performance. Considering the increasing number of tDCS studies and the high relevance of bimanual motor skills the purpose of this review article is to provide a preliminary systematic characterization of tDCS effects on bimanual actions.

## Review Criteria

The present review article focuses on studies addressing tDCS effects explicitly on bimanual motor performance, thus investigating bimanual motor outcome was the decisive criterion. To this end, a computer-based search of PubMed and Science Direct for articles from 2000 to 2017 was carried out in August 2017 using the following keywords: “bimanual” AND “transcranial direct current stimulation” OR “tDCS” OR “transcranial electrical stimulation” OR “non-invasive brain stimulation” OR “transcranial stimulation” and “coordination” AND “transcranial direct current stimulation” OR “tDCS” OR “transcranial electrical stimulation” OR “non-invasive brain stimulation” OR “transcranial stimulation”. A total of 18 articles matched the criteria. Five articles did not directly investigate bimanual motor skills and were therefore excluded.

## Transcranial Direct Current Stimulation

TDCS allows the modulation of neural excitability (Bindman et al., 1962, 1964; Nitsche and Paulus, 2001). Using intensities of 0.5–2 milliampere (mA), a constant current is applied to the cerebral cortex via saline soaked sponges or gel-electrodes through the skull. Two electrodes with a surface area of 15–35 cm^2^ are used mostly, resulting in a current density of 0.014–0.133 mA/cm^2^ (Ho et al., 2016; Woods et al., 2016). In high-definition tDCS (HD-tDCS), smaller (1–5 cm^2^) and often more than two electrodes (e.g., 4 × 1 configuration) are used to increase the current density and focality of the stimulated area (Villamar et al., 2013; Alam et al., 2016). Although its exact physiological mechanisms are still under debate, evidence exists that anodal tDCS increases the neural excitability of the stimulated area, while cathodal tDCS decreases it. Anodal stimulation shifts the resting membrane potential closer to the critical depolarization threshold, resulting in a higher excitability and spiking rate, while the opposite effect of tonic hyperpolarization is associated with cathodal stimulation (Bindman et al., 1962; Nitsche and Paulus, 2000; Romero Lauro et al., 2014). These effects have mainly been derived from the stimulation of the primary motor cortex (M1), although comparable effects have also been demonstrated in visual (Antal et al., 2004; Accornero et al., 2007), somatosensory (Dieckhöfer et al., 2006), and auditory cortices (Zaehle et al., 2011). Nevertheless, anatomical differences—in particular, the spatial orientation of neurons—need to be considered in order to understand the effects of tDCS on other brain areas (Accornero et al., 2007). Moreover, the effects of tDCS depend on several factors like current intensity, duration and timing of tDCS relative to the specific task (prior to vs. during; Stagg et al., 2011; Batsikadze et al., 2013; Monte-Silva et al., 2013). The shape, size, number, and positions of the electrodes also determine the characteristics of tDCS such as distribution of the induced electrical field (Ho et al., 2016; Naros et al., 2016; Woods et al., 2016). Individual attributes of the participants (e.g., head anatomy, age) but also the excitability of the stimulated area are thought to influence the effects of tDCS yielding intra- and inter-individual variability on physiological and behavioral measures (Ridding and Ziemann, 2010; Li et al., 2015; Opitz et al., 2015). Besides local effects on the stimulated brain area, tDCS is presumed to affect the excitability of functionally connected areas as well. Such remote effects can be in the same (Antal et al., 2011) or opposite (Stagg et al., 2009) direction as the effects in the stimulated area.

Changes of M1 excitability associated with tDCS have been found to persist after cessation of stimulation (Nitsche and Paulus, 2000, 2001). Such after-effects are associated with alterations in N-methyl-d-aspartate (NMDA) receptors (Liebetanz et al., 2002)—at least within M1—yielding long-term potentiation (LTP)-like mechanisms. In motor learning, these effects seem to be strongest when tDCS is co-applied with motor training (Reis and Fritsch, 2011; Stagg et al., 2011) and applied over multiple days (Reis et al., 2009; Alonzo et al., 2012; Saucedo Marquez et al., 2013).

## tDCS and Bimanual Motor Skills in Healthy Volunteers

Bimanual motor skills require a well-coordinated interplay between the upper limbs. Multiple brain areas are involved in orchestrating bimanual movements in space and time. Because the supplementary motor area (SMA) plays a pivotal role in such tasks (Swinnen, 2002; Debaere et al., 2004; Swinnen and Wenderoth, 2004; Swinnen and Gooijers, 2015), Carter et al. (2015) examined the effects of tDCS applied over the SMA on the stability, consistency, and transition of metronome-paced bimanual forearm movements from in- to anti-phase movements and vice versa. They reported improved stability and consistency as well as a delayed spontaneous transition from the anti- to the more stable in-phase pattern following anodal tDCS, while cathodal tDCS did not affect task performance (Carter et al., 2015). In line with this observation, anodal tDCS of the SMA was found to be associated with faster intentional switches from anti- to in-phase movements (Carter et al., 2017). These findings underline a central role of the SMA in the control of bimanual movements and suggest that tDCS represents a suitable method for the transient modulation of this process.

Typing a text on a keyboard requires bilateral well-coordinated, skilled finger movements, and particularly practice to achieve successful task performance. Gomes-Osman and Field-Fote (2013) investigated the effects of anodal tDCS over bilateral M1 on a bimanual typing task. Task performance was assessed immediately before and after stimulation. After five consecutive training days, participants showed a larger improvement in the number of correctly-typed bimanual sequences following anodal tDCS as compared to sham stimulation. However, this effect vanished after a 1-week retention interval. Ciechanski and Kirton (2017) examined the effects of tDCS on dexterity using the Purdue Pegboard Test (PPT; Tiffin and Asher, 1948). The PPT measures uni- and bimanual hand functions such as placing the maximum number of pegs in the pegboard within 30 s. While the bimanual task requires symmetrical movements of both hands, a second bimanual task (assembly) requires the asymmetrical (alternate) use of both hands to build a maximum number of small assemblies consisting of pins, collars and washers within 60 s (Desrosiers et al., 1995). Ciechanski and Kirton (2017) reported improved bimanual motor performance in healthy school children after training of the left hand with concurrent anodal tDCS applied to the contralateral M1 corresponding to C4. Interestingly, this effect was also present when cathodal tDCS was applied to the ipsilateral M1 (C3), but not after sham tDCS. While the symmetrical bimanual performance was facilitated, the more complex (asymmetrical) “assembly test” was not affected by either stimulation. After a retention interval of 6 weeks, improved task performance remained stable, suggesting enhanced motor consolidation associated with tDCS. Pixa et al. (2017b) also used the PPT to investigate the effect of multichannel HD-tDCS targeting bilateral M1 (see Table 1 for electrode positions). After a training period of 3 days with concurrent stimulation, larger cumulative performance gains in participants receiving anodal HD-tDCS compared to the sham-stimulated control group were found. Improved performance was indicated for the unimanual task of the dominant right hand and the bimanual PPT. Performance gains remained stable over a 1-week retention interval and further improvement was found for right-hand performance. As compared to sham tDCS, no significant differences were found for the unimanual left-hand task and—again—the more complex bimanual “assembly” task was not affected by tDCS (Pixa et al., 2017b). The same stimulation protocol was used to investigate effects of bilateral anodal HD-tDCS on the performance of a complex sequential bimanual stacking task (Pixa et al., 2017a). After 3 days of training in sport stacking (cup stacking) with concurrent anodal HD-tDCS, faster stacking performance compared to the sham group was found. This effect occurred for only one of the two required stacking formations (3-6-3), while only a statistical trend emerged for the more complex task-version (1-10-1). Re-testing after 1 week revealed sustained superior performance in the 3-6-3 stack for the anodal HD-tDCS group.

**Table 1:** Overview of studies investigating the effects of transcranial direct current stimulation (tDCS) on bimanual motor skills in healthy volunteers.

Author(s)	Stimulation		Intensity (mA)	Duration (min)	Timing	Frequency	Sample	Task	Outcome	Control
	Anode(s)	Cathode(s)								
Carter et al. (2015)	SMA (1.8 cm anterior to Cz)	Center of forehead	1	10	Between pre- and post blocks	Single session 1 day	10 Healthy	Metronome paced rhythmic supination-pronation bimanual movements	Increased stability and accuracy in anti-phase patterns following anodal tDCS; No effect of cathodal tDCS	Sham
	Center of forehead	SMA (1.8 cm anterior to Cz)								
Carter et al. (2017)	SMA (1.8 cm anterior to Cz)	Center of forehead	1	10	Between pre- and post blocks	Single session 1 day	10 Healthy	Metronome paced rhythmic supination-pronation bimanual movements	Faster intentional switching from anti- to in-phase following anodal tDCS	Sham
	Center of forehead	SMA (1.8 cm anterior to Cz)								
Ciechanski and Kirton (2017)	C4	Contralateral supraorbital	1	10	Concurrent with training	Single session multiple days (3 days)	24 Healthy (children)	PPT	Enhanced performance in PPT except for assembly. Improvements sustained after 6 weeks	Sham
	Contralateral supraorbital	C3	1 or 2							
Furuya et al. (2013)	C3	C4	1	24	Concurrent with training	Single session 1 day	12 Healthy (expert pianists)	Metronome paced bimanual symmetrical sequential typing task	No improvement	Sham
	C4	C3								
Furuya et al. (2014a)	C3	C4	2	15	Between pre- and post blocks	Single session 1 day	26 Healthy (13 pianists) (13 novices)	Metronome paced bimanual symmetrical sequential typing task	Improved performance in novices, slight deterioration in expert pianists	Sham
	C4	C3								
Gomes-Osman and Field-Fote (2013)	C3 & C4	Bilateral supraorbital	1	20	Between pre- and post blocks	Single session multiple days (5 days)	28 Healthy	Bimanual asymmetrical sequential typing task	Anodal tDCS improved bimanual typing performance. This effect vanished after 1 week	Sham
McCambridge et al. (2016)	C4	C3	1	15	Between pre- and post blocks	Single session 1 day	16 Health	Metronome paced anticlockwise bimanual circle tracing task	No improvements in bimanual performance following tDCS. Slightly increased performance after sham	Sham
Pixa et al. (2017a)	C1 & C2	FC5, CP5, T7 & FC6, CP6, T8	1	15	Concurrent with training	Single session multiple days (3 days)	32 Healthy	Bimanual asymmetrical sequential stacking task (cup stacking)	Selectively enhanced performance. Improvements sustained after 1 week.	Sham
Pixa et al. (2017b)	C1 & C2	FC5, CP5, T7 & FC6, CP6, T8	1	15	Concurrent with training	Single session multiple days (3 days)	31 Healthy	PPT	Enhanced performance on right and bimanual PPT except for assembly. Improvements sustained after 1 week.	Sham
Vancleef et al. (2016)	L M1	Contralateral supraorbital	2	20	Concurrent with training	Single session multiple days (4 days)	75 Healthy	BTT	No improvement	Sham
	L DLPFC (F3)									

*BTT, bimanual tracking task; DLPFC, dorsolateral prefrontal cortex; EEG positions according to international 10–10 EEG system; L, left; M1, primary motor cortex; PPT, Purdue pegboard test; SMA, supplementary motor area*.

In two studies, Furuya et al. (2013, 2014a) demonstrated that the effects of tDCS on bimanual motor performance depend on the subjects’ level of expertise. While highly-trained expert pianists did not benefit from bilateral tDCS over M1 (anode right M1 and cathode left M1 and vice versa) in a bimanual finger typing task, the analysis revealed that only pianists who commenced piano training at an advanced age showed selectively improved performance following tDCS, irrespective of the stimulation protocol (Furuya et al., 2013). A second study using the same bimanual task, suggests that musically untrained volunteers (novices) significantly improved bimanual performance following tDCS, whereas expert pianists did not. Rather, the skilled finger movements of the pianists were found to be slightly deteriorated after verum stimulation compared to sham stimulation (Furuya et al., 2014a).

It should also be stressed that other studies have failed to provide evidence of significant stimulation effects on bimanual task performance. McCambridge et al. (2016) applied bilateral tDCS with the anode over the right M1 and the cathode over the left M1 and assessed the effects on left-hand and bimanual circle tracing performance. The results indicated a marginal effect on the bimanual task in low performers who received sham stimulation, while no significant effect was found in participants who received active tDCS. Vancleef et al. (2016) examined the effects on task performance of anodal tDCS applied to the left M1 or the left dorsolateral prefrontal cortex (DLPFC) during training on a complex bimanual tracking task (BTT). The BTT requires tracking of a moving dot by rotating two dials with the left (vertical direction) and right (horizontal direction) hands in different frequency-ratios. The authors reported improved motor performance for all participants, independent of tDCS.

Despite the heterogeneity in findings, the data begin to reveal that tDCS may affect relatively easy rather than complex bimanual tasks in particular and that it appears to be more effective in novice volunteers rather than in experts.

## tDCS and Bimanual Motor Performance in Patients

Impairment of bimanual skills can occur due to a wide variety of neurological diseases, like stroke or Parkinson’s disease, and task performance might be influenced by altered cortical excitability, such as in focal dystonia (FD). In FD, the affected M1 shows pathologically increased activity that is associated with involuntary movements (e.g., tremor) and muscle spasms of the contralateral effector (Cohen and Hallett, 1988; Stinear and Byblow, 2004). Since tDCS likely modulates motor cortical excitability, Furuya et al. (2014b) investigated the effects of five different stimulation configurations (see Table 2 for an overview of different electrode montages) on bimanual task performance in pianists suffering from FD of the right hand and in healthy controls. The study revealed that bilateral tDCS over M1 (affected left M1; cathode over C3, anode over C4) led to increased accuracy of the dystonic hand, both during and after tDCS, while performing metronome-paced symmetrical bimanual finger movements. This effect was not found for bilateral tDCS with the anode over C3, unilateral tDCS (anode over C4), sham stimulation or—noteworthy for bilateral tDCS—with the cathode over C3 and anode over C4 without concurrent bimanual training. Interestingly, performance gains were positively correlated with FD severity.

**Table 2:** Overview of studies investigating the effects of tDCS on bimanual motor skills in patients.

Author(s)	Stimulation		Intensity (mA)	Duration (min)	Timing	Frequency	Sample	Task	Outcome	Control
	Anode(s)	Cathode(s)								
Furuya et al. (2014b)	C3	C4	1	24	Concurrent with training	Single session 1 day	20 (10 pianists with FD right hand) (10 healthy pianists)	Metronome paced bimanual symmetrical sequential typing task	Improved fine motor control indicated by decreased variability in rhythmic keystrokes performed by the affected hand during and after cathodal tDCS. No effect on finger tapping task. No effect in heathy controls.	Sham
	C4	C3								
	C4	Contralateral supraorbital								
Middleton et al. (2014)	ipsilesional M1	Contra-lesional M1	1.5	15	Concurrent with physical therapy	Single session multiple days (24 days)	5 (stroke or TBI patients)	PPT and OHT	Enhanced bimanual performance on the object hit task. No improvement in fine manual dexterity (PPT).	Sham
Takeuchi et al. (2012)	ipsilesional M1	Contra-lesional supraorbital	1	20	Prior to training	Single session 1 day	27 (stroke patients)	Metronome paced bimanual antiphase finger tapping task	Less deterioration in antiphase finger to thumb tapping, when co-applied to low-frequency rTMS.	Sham

*EEG positions according to international 10–10 EEG system; FD, focal dystonia; M1, primary motor cortex; OHT, object hit task; PPT, Purdue pegboard test; TBI, traumatic brain injury*.

Traumatic brain injuries (TBI) can also impair bimanual motor task performance. In a pilot study, Middleton et al. (2014) combined bihemispheric tDCS with physical therapy of the upper extremities in patients suffering from stroke and TBI. Bihemispheric tDCS was applied concurrently with physical therapy for 15 min, with the anode over the ipsilesional M1. The intervention was implemented over 24 sessions, with three sessions per week. The results revealed improved function of the upper extremities in clinical (Fugl-Meyer Assessment UE, Box and Block Test, PPT, Stroke Impact Scale) and robotic measures (Visually Guided Reaching Task, Object hit task (OHT)). Focusing on bimanual measures, performance in the OHT showed improved bimanual coordination, indicating superior gross motor function. However, bimanual fine motor control in the PPT did not benefit from stimulation. The observed gains persisted up to 6 months. In line with Middleton et al. (2014) the data support the notion that tDCS represents a feasible approach facilitating the effects of physical therapy of the upper extremities. Takeuchi et al. (2012) combined low-frequency, single-pulse, repetitive transcranial magnetic stimulation (rTMS) over the unaffected hemisphere of stroke patients with simultaneous application of anodal tDCS over the affected M1. The combination of rTMS and anodal tDCS was found to reduce motor impairment in these patients (Takeuchi et al., 2012).

## Discussion

In the light of the abundance of everyday tasks requiring bimanual actions and their importance in independent living, the purpose of this review article was to summarize the few existing studies investigating the effects of tDCS on bimanual motor skills. The data suggest that—although some studies have failed to show tDCS-specific effects on bimanual tasks—tDCS has the potential to enhance bimanual motor performance in healthy volunteers as well as in patients suffering from neurological diseases. The overview reveals that—besides the well-known inter- and intra-individual variability of outcome-measures, conflicting study results may be attributed to substantial variations in stimulation protocols, bimanual tasks, and study designs (see Tables 1, 2). Nevertheless, this review article provides a starting point for a systematic evaluation of tDCS effects on bimanual task performance.

Taken together, the data indicate that M1 was the preferred brain area for tDCS application to modulate bimanual performance, in line with the majority of tDCS studies addressing unimanual tasks (for an overview see Buch et al., 2017). However, it must be stressed that the use of stimulation protocols commonly applied for unimanual tasks might represent an oversimplification of the brain processes subserving bimanual task performance since brain areas involved and—more importantly—the temporally precise functional interaction between these areas are assumed to differ between bi- and unimanual tasks (Debaere et al., 2004; Swinnen and Wenderoth, 2004; Wenderoth et al., 2005; Pollok et al., 2007). Several brain regions which are involved in bimanual motor performance are accessible by means of tDCS. The parietal cortex (PC) is suggested to play a pivotal role in bimanual performance through multisensory integration and guidance of movements (Battaglia-Mayer et al., 2006; Buneo and Andersen, 2006), and, learning of bimanual skills (Debaere et al., 2004). Additionally, the right superior temporal gyrus (STG) is proposed to be causally involved in monitoring bimanual spatio-temporal goals (Duque et al., 2010). Furthermore, until now no study had investigated effects of premotor cortex (PMC) or cerebellar tDCS on bimanual motor skills. Moreover, different task demands such as the required type of bimanual action (e.g., symmetric or asymmetric) to achieve a specific task-goal, as well as individual expertise (Furuya et al., 2014a), are likely related to distinct activation patterns within the motor network of bimanual actions (Puttemans et al., 2005; Jantzen et al., 2008; Duque et al., 2010; Whitall et al., 2011). Since complex bimanual tasks are associated with brain activation extending towards the prefrontal, parietal and temporal areas (Gross et al., 2002; Debaere et al., 2004; Hardwick et al., 2013; Swinnen and Gooijers, 2015), the stimulation of a particular brain area might not be effective in modulating complex bimanual skills. This hypothesis fits well with the observation that more complex bimanual tasks remain unaffected by tDCS (Vancleef et al., 2016; Ciechanski and Kirton, 2017; Pixa et al., 2017a,b). Since the neural mechanisms of the wide variety of bimanual actions, as well as the neurophysiological mechanisms underlying tDCS, are not completely understood, future studies need to consider neurophysiological measures using, e.g., TMS, electroencephalography (EEG), magnetoencephalography (MEG) or functional near-infrared spectroscopy (fNIRS). This is particularly important since intra- and inter-individual variability in responses to tDCS is suggested to highly influence study outcomes (Li et al., 2015).

So far, only three studies were identified that have investigated the effects of tDCS on bimanual skills in patients. Although these studies widely differ in terms of the respective stimulation protocol and—even more important—the underlying disease, the findings suggest facilitating effects of tDCS on bimanual task performance. Although sparse, the data imply that tDCS in combination with motor training represents a suitable method for neuro-rehabilitation.

Finally, besides tDCS other techniques like transcranial alternate current stimulation (tACS) or transcranial random noise stimulation (tRNS) are suitable methods for the non-invasive modulation of brain processes subserving motor learning (Prichard et al., 2014; Pollok et al., 2015). However, to the best of our knowledge, no study that had adopted one of these methods in bimanual tasks has been published until yet.

## Conclusion

Up until now, knowledge about the effects of tDCS on bimanual performance has remained limited due to a relatively small number of studies with mixed results. However, despite the heterogeneity in stimulation protocols, study designs, and paradigms, the data suggest that tDCS has the potential to enhance bimanual motor performance in healthy volunteers as well as in patients suffering from a variety of neurological diseases. Noteworthy, the data do not allow the identification of specific tDCS parameters as most effective to modulate bimanual motor skills. Therefore, tailoring tDCS protocols to bimanual motor tasks will critically challenge future studies.

## Author Contributions

NHP contributed to the conceptualization of the review, performed the literature research, interpretation of the data, wrote the manuscript, approved the final version of the manuscript and acted as corresponding author. BP substantially contributed to the data interpretation, wrote parts of the manuscript and critically revised the manuscript.

## Conflict of Interest Statement

The authors declare that the research was conducted in the absence of any commercial or financial relationships that could be construed as a potential conflict of interest.
